# Association of schizophrenia with fracture‐related femoral neck displacement: A cross‐sectional retrospective study

**DOI:** 10.1002/pcn5.195

**Published:** 2024-05-06

**Authors:** Yukiyo Inoue, Akihiro Tokushige, Takeshi Kinjyo, Shinichiro Ueda

**Affiliations:** ^1^ Department of Psychiatry Takamatsu Red Cross Hospital Takamatsu‐Shi Kagawa Japan; ^2^ Department of Clinical Research and Management University of Ryukyus Graduate School of Medicine Nishihara‐cho Okinawa Japan; ^3^ Department of Clinical Pharmacology & Therapeutics University of Ryukyus Graduate School of Medicine Nishihara‐cho Okinawa Japan; ^4^ Department of Orthopedics Okinawa Prefectural Nanbu Medical Center & Children's Medical Center Heabaru‐cho Okinawa Japan

**Keywords:** displacement, femoral neck fracture, garden classification, schizophrenia

## Abstract

**Aim:**

Fracture‐related femoral neck displacement is more likely in patients with schizophrenia because of delayed diagnosis, as these patients frequently have less severe fracture‐associated subjective symptoms. This study aimed to investigate the association of schizophrenia with the risk of fracture‐related femoral neck displacement in hospitalized patients.

**Methods:**

We retrospectively analyzed the medical records of patients with femoral neck fractures treated between April 2013 and March 2018 at a single institution. Multivariate logistic regression was used to explore the relationship between schizophrenia and fracture‐related femoral neck displacement after adjusting for risk factors.

**Results:**

We compared 30 and 194 patients with and without schizophrenia, respectively. The prevalence of fracture‐related displacement was 80.0% in patients with schizophrenia and 62.4% in the controls (*p* = 0.06). After adjusting for confounding variables, schizophrenia significantly correlated with fracture‐related femoral neck displacement (odds ratio: 4.74, 95% confidence interval: 1.09–20.60, *p* = 0.0378).

**Conclusions:**

Schizophrenia is associated with a higher risk of severe femoral neck fracture. To improve outcomes and alleviate the societal burden of femoral neck fractures, early radiographic assessment and surgical intervention for femoral fractures are essential for patients with schizophrenia, even in those without pain symptoms.

## Graphical abstract

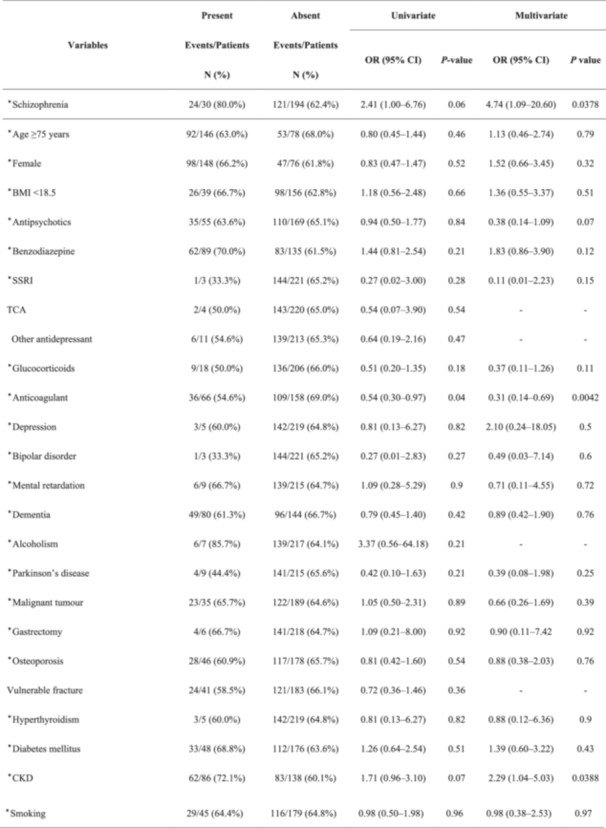

## INTRODUCTION

In Japan, which has one of the world's largest older populations, the number of femoral neck fractures is expected to increase.^1^ Besides increased medical costs, older patients with femoral neck fractures incur social consequences, such as increased mortality, decreased quality of life, increased need for nursing care, and an inability to return to their original living environment due to decreased mobility and walking ability.^2^ Treatment principles for femoral neck fractures include early surgery, weaning, and functional training.^2^, ^3^ Surgical options for femoral neck fractures include internal fixation and arthroplasty. In patients without displacement (less severe) and with displacement (more severe), internal fixation and hip arthroplasty, respectively, are the procedures of choice.^2^ Internal fixation causes fewer complications^3^ and a smaller postoperative social impact. However, even nondisplaced fractures may become displaced over time; therefore, internal fixation should be performed before such displacement occurs.

Schizophrenia is one of the most severe psychiatric disorders worldwide, and can cause many physical complications.^4^ Several studies have reported that patients with schizophrenia are at a higher risk of osteoporotic fractures.^5^, ^6^, ^7^, ^8^, ^9^, ^10^ One possible explanation for the higher risk of fractures in schizophrenic patients is that antipsychotic‐related hyperprolactinemia leads to impaired bone cell metabolism and a high percentage of bone mineral density loss.^9^, ^11^, ^12^, ^13^, ^14^, ^15^, ^16^, ^17^, ^18^, ^19^, ^20^ A second possible explanation for the higher risk of bone fractures in patients with schizophrenia is that these patients are more likely to fall because of the side‐effects of antipsychotic medications: sedation, extrapyramidal symptoms, and orthostatic hypotension.^13^, ^17^, ^18^, ^21^

Femoral neck fractures usually cause severe pain that makes walking difficult. However, in clinical practice, patients with schizophrenia and femoral neck fractures often walk without pain, and when the fracture is identified, it is displaced. Though femoral neck fractures in patients with schizophrenia are associated with a risk of high severity, few studies have focused on the relationship between the severity of femoral neck fractures and schizophrenia. In this study, we aimed to clarify whether schizophrenia is a risk factor for fracture‐related femoral neck displacement.

## METHODS

### Study setting and informed consent

The Okinawa Prefectural Nanbu Medical Center and Children's Medical Center (NMC) are public general hospitals with 46 clinical departments and emergency medical care centers. The psychiatric department has Medical Psychiatric Units, consisting of five beds, that admit patients with severe physical complications. The NMC is a designated referral hospital for physical complications for the entire prefectural medical territory, and patients who have been hospitalized in other regional psychiatric facilities and have developed diseases or injuries are frequently transferred to the NMC.

This study was approved by the Ethics Committee of the NMC and was conducted in accordance with the ethical guidelines for clinical research of the Ministry of Health, Labour, and Welfare, Japan, and the Declaration of Helsinki. Informed consent was obtained from all participants on an opt‐out basis by in‐hospital and website postings. This study dealt with clinical records at NMC, and to ensure the accuracy of the data, we conducted a data review.

### Study design and participants

Data were retrospectively collected from the medical records of patients who had completed medical care. Initially, from among patients who were hospitalized in NMC from April 1, 2013 to March 31, 2018, we collected clinical records in which “code 7200” (“femoral neck fracture” in the *International Statistical Classification of Diseases and Related Health Problems*, 10th Revision [ICD‐10]^22^) was registered retrospectively. This code is input to recommend an X‐ray examination at the patient's first visit; therefore, fracture‐suspected cases were included. Next, an orthopedic specialist, a member of our research group, reviewed the simple radiographs of the collected patients to assess femoral neck fractures and their Garden classification. The patients' personal information (name, age, and sex) on the simple radiographs and the date of imaging were concealed to ensure blinding.

**Figure 1:**
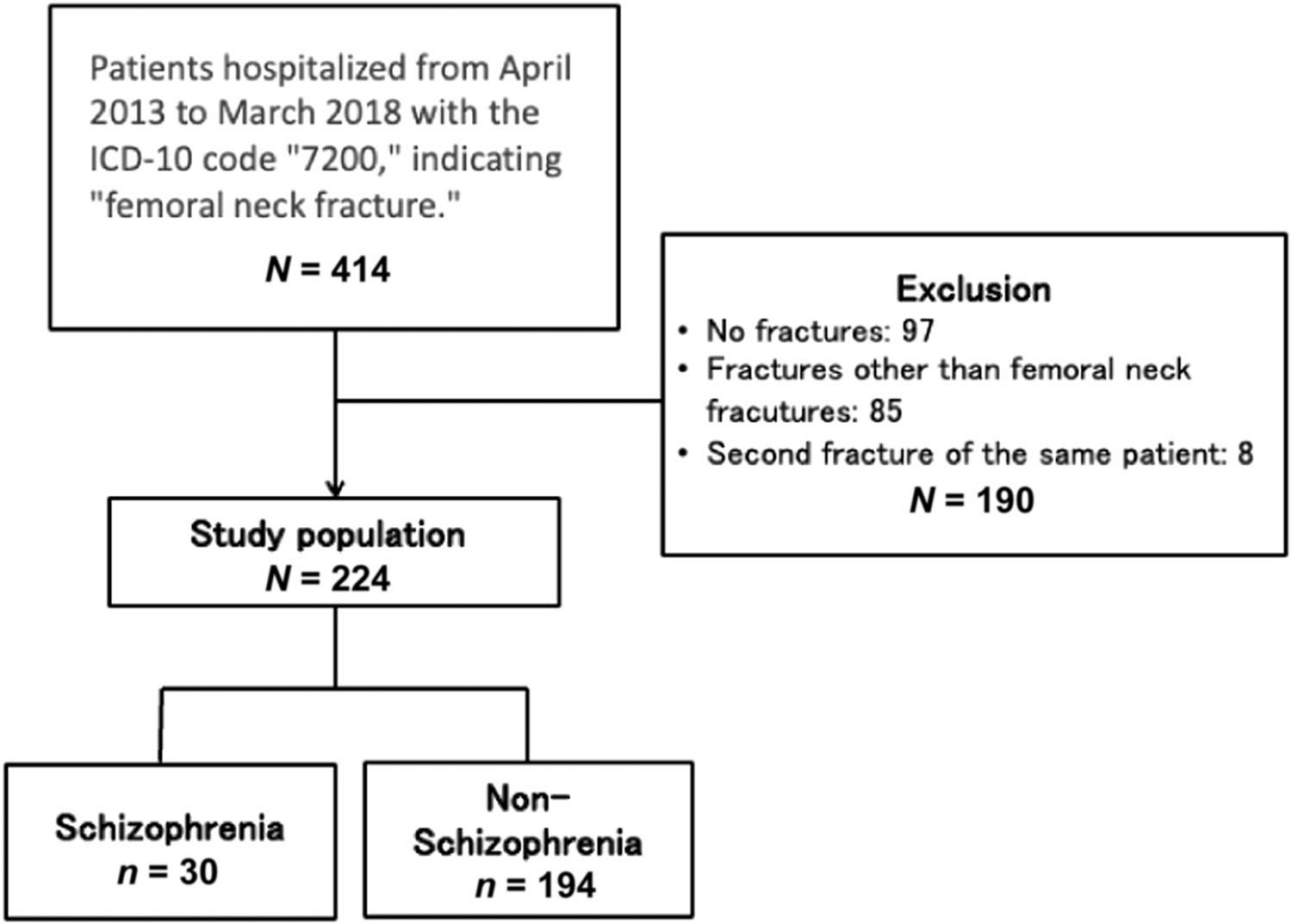
Flowchart of the participant selection and enrolment in the study.

Patients diagnosed with femoral neck fracture were enrolled in this study. Patients with no fractures, fractures other than femoral neck fractures, second‐time fractures in the same patient, and those who refused research cooperation were excluded. As shown in Figure 1, 414 patients had been registered with code 7200 during the survey period. After excluding 97 patients without fractures, 85 with fractures other than femoral neck fractures, and eight with second‐time fractures in the same patient, 224 participants were enrolled. No patient refused to participate. We stratified the 224 patients according to the absence or presence of schizophrenia.

### Outcome measures

The primary outcome was the severity of femoral neck fractures (Garden classification). The Garden classification is a widely accepted method for describing femoral neck fractures. The Garden I/II category was classified as no displacement, and Garden III/IV as displacement (Figure S1).^23^

### Definitions of variables

The NMC psychiatry department diagnoses mental illness based on the ICD‐10^22^ or the *Diagnostic and Statistical Manual of Mental Disorders* (4th edition)^24^. Among patients for whom the NMC psychiatry department was not involved in the diagnosis, we adopted the original diagnoses from previous psychiatric hospitals.

As no previous study has used variables for studying the risk of displacement, we employed variables for the risk of femoral neck fracture. The survey variables included age, sex, and BMI, as well as risk factors for femoral neck fracture that have been reported in the Japanese Orthopedic Association practice guidelines (in Japanese) and could be confirmed in the medical records: use of psychotropic drugs, glucocorticoids, and anticoagulants (including antiplatelet drugs); and patient's medical history (PMH) of mental disorder, diabetes mellitus, malignant tumors, gastrectomy, osteoporosis, vulnerable fracture, hyperthyroidism, chronic kidney disease (CKD), and/or smoking. Furthermore, we checked for Parkinson's disease, which has been studied in relation to the non‐displacement of intertrochanteric fractures previously.^23^

Psychotropic drugs were subcategorized into antipsychotics, benzodiazepines, selective serotonin reuptake inhibitors (SSRIs), and tricyclic antidepressants (TCAs). Mental disorders were subcategorized into schizophrenia, depression, bipolar disorder, mental retardation, dementia, and alcoholism. “Use” of medicine was defined as “taking medicine when hospitalized.” The categorization criteria for mental disorders are as mentioned above (“Definitions of variables”). In addition, based on the information in clinical records, mental retardation included “students and graduates of special‐needs schools.” Dementia included patients who “scored 20 or less on the revised Hasegawa Dementia Scale and 23 or less on the Mini‐Mental State Examination” or who were “taking drug therapy.” Alcoholism included “PMH of alcoholism” or “users of self‐help group.” Physical disorder was categorized as follows, based on the information in clinical records: malignant tumor; “PMH of treatment for malignant tumor,” gastrectomy; “PMH of gastrectomy,” osteoporosis; “taking drug therapy,” vulnerable fracture; “PMH of contralateral hip, vertebral, proximal humerus and distal radius fractures,” diabetes mellitus; “glycated hemoglobin (HbA1c) 6.5% or more” or “blood glucose 200 mg/dL or more” or “taking drug therapy,” hyperthyroidism; “treatment history for drugs, isotope, or surgery,” CKD; “estimated glomerular filtration rate less than 60 mL/min/1.73 m^2^,” “after introduction of maintenance dialysis” or “after kidney transplant,” smoking; “habitual smoking regardless of a non‐smoking term,” Parkinson's disease; “PMH of Parkinson's disease” or “taking drug therapy.”

### Statistical analysis

We compared details using Pearson's chi‐square test or Fisher's exact test for categorical variables and the independent Student's *t*‐test or Mann–Whitney *U*‐test for continuous variables, where appropriate. Nominal variables are presented as numbers and percentages and continuous variables as median with interquartile range (IQR).

To investigate the association between displacement and schizophrenia, we calculated a univariate and multivariate logistic regression model. In the multivariate model, we adjusted for the clinically relevant factors associated with displacement, as the following variables described in the “Definitions of variables” section: age; sex; BMI; antipsychotics; benzodiazepines; SSRIs; TCAs; other antidepressants; glucocorticoids; anticoagulants; schizophrenia; depression; bipolar disorder; mental retardation; dementia; alcoholism; Parkinson's disease; malignant tumors; gastrectomy; osteoporosis; vulnerable fractures; hyperthyroidism; diabetes mellitus, CKD; and smoking. All statistical analyses were two‐sided, and a *p* < 0.05 was considered statistically significant. All analysis was conducted using JMP Pro13.1.0 (SAS Institute Inc.).

## RESULTS

### Baseline characteristics

As shown in Table 1, 30 cases of femoral neck fracture in patients with schizophrenia were detected and compared with 194 controls. Participants in the schizophrenia group were significantly younger than controls (66.0 ± 8.8 vs. 78.4 ± 11.9 years, *p* < 0.001); 73.3% of the schizophrenia group and 65.0% of the control group were women, though no sex‐related significant difference was noted. The schizophrenia group had a higher proportion of antipsychotic or benzodiazepine use, and the proportion of anticoagulant use was higher in controls. Regarding mental disorders, the proportion of mental retardation was higher in patients with schizophrenia, and the proportion of dementia was higher in controls. Among PMHs with physical disorders, the proportion of patients with CKD was higher in the control group. There was no significant intergroup difference in smoking status.

**Table 1:** Baseline characteristics of patients with femoral neck fracture, stratified by the presence or absence of schizophrenia.

Variables	All patients	Schizophrenia	Non‐schizophrenia	*p* value
	*N* = 224	*N* = 30	*N* = 194	
Age (years)	76.8 ± 12.3	66.0 ± 8.8	78.4 ± 11.9	<0.001
Age ≥75 years	146 (65.2%)	5 (16.7%)	141 (72.7%)	<0.001
Female	148 (66.1%)	22 (73.3%)	126 (65.0%)	0.37
BMI (kg/m^2^)	21.6 ± 3.7	21.0 ± 3.3	21.7 ± 3.7	0.32
BMI < 18.5 (kg/m^2^)	39 (20.0%)	7 (25.9%)	32 (19.1%)	0.41
Medications				
Antipsychotics	55 (24.6%)	26 (86.7%)	29 (15.0%)	<0.001
Benzodiazepine	89 (39.7%)	24 (80.0%)	65 (33.5%)	<0.001
SSRI	3 (1.3%)	1 (3.3%)	2 (1.0%)	0.35
TCA	4 (1.8%)	0 (0.0%)	4 (2.1%)	1
Other antidepressant	11 (4.9%)	2 (6.7%)	9 (4.6%)	0.65
Glucocorticoids	18 (8.0%)	2 (6.7%)	16 (8.3%)	1
Anticoagulant	66 (29.5%)	3 (10.0%)	63 (32.5%)	0.012
Mental disorder				
Depression	5 (2.2%)	0 (0.0%)	5 (2.6%)	1
Bipolar disorder	3 (1.3%)	0 (0.0%)	3 (1.6%)	1
Mental retardation	9 (4.0%)	4 (13.3%)	5 (2.6%)	0.021
Dementia	80 (35.7%)	2 (6.7%)	78 (40.2%)	0.0004
Alcoholism	7 (3.1%)	0 (0.0%)	7 (3.6%)	0.6
Medical history				
Parkinson's disease	9 (4.0%)	0 (0.0%)	9 (4.6%)	0.61
Malignant tumor	35 (15.6%)	4 (13.3%)	31 (16.0%)	1
Gastrectomy	6 (2.7%)	0 (0.0%)	6 (3.1%)	1
Osteoporosis	46 (20.5%)	4 (13.3%)	42 (21.7%)	0.29
Vulnerable fracture	41 (18.3%)	5 (16.7%)	36 (18.6%)	0.8
Hyperthyroidism	5 (2.2%)	0 (0.0%)	5 (2.6%)	1
Diabetes mellitus	48 (21.4%)	5 (16.7%)	43 (22.2%)	0.49
CKD	86 (38.4%)	5 (16.7%)	81 (41.8%)	0.009
Smoking	45 (20.1%)	9 (30.0%)	36 (18.6%)	0.15

Abbreviations: BMI, body mass index; CKD, chronic kidney disease; SSRI, selective serotonin‐reuptake inhibitor; TCA, tricyclic antidepressant.

### Comparison of displacement ratio

As shown in Table 2, 145/224 (64.7%) fractures were displaced in the study population, including 24/30 (80.0%) in the schizophrenia group and 121/194 (62.4%) in the control group, without significant intergroup difference.

**Table 2:** Displacement of femoral neck fracture, stratified by the presence or absence of schizophrenia.

Variables	All patients	Schizophrenia	Non‐schizophrenia	*p* value
	*N* = 224	*N* = 30	*N* = 194	
Displacement	145 (64.7%)	24 (80.0%)	121 (62.4%)	0.06

*Note*: Displacement: Garden 3 and 4.

### Association of schizophrenia with displacement of femoral neck fracture

In this study, schizophrenia was analyzed as the main factor and the others as adjustment factors. Table 3 shows the displacement‐related factors in the multivariate logistic regression model analysis. In univariate analysis, only use of anticoagulants showed significance (odds ratio [OR]: 0.54, 95% confidence interval [CI]: 0.30–0.97, *p* = 0.040); schizophrenia was a trend, though not significant statistically (OR: 2.41, 95% CI: 1.00–6.76, *p* = 0.06). However, after adjusting for clinically relevant factors in multivariate analysis, we found three factors that showed significance: schizophrenia (OR: 4.74, 95% CI: 1.09–20.60, *p* = 0.0378), use of anticoagulants (OR: 0.31, 95% CI: 0.14–0.98, *p* = 0.0042), and CKD (OR: 2.29, 95% CI: 1.04–5.03, *p* = 0.0388).

**Table 3:** Independent risk factors for displacement of femoral neck fracture based on multiple logistic regression.

Variables	Present Events/patients	Absent Events/patients	Univariate		Multivariate	
	*N* (%)	*N* (%)	OR (95% CI)	*p*‐value	OR (95% CI)	*p* value
Schizophrenia^a^	24/30 (80.0%)	121/194 (62.4%)	2.41 (1.00–6.76)	0.06	4.74 (1.09–20.60)	0.0378
Age ≥ 75 years^a^	92/146 (63.0%)	53/78 (68.0%)	0.80 (0.45–1.44)	0.46	1.13 (0.46–2.74)	0.79
Female^a^	98/148 (66.2%)	47/76 (61.8%)	0.83 (0.47–1.47)	0.52	1.52 (0.66–3.45)	0.32
BMI < 18.5^a^	26/39 (66.7%)	98/156 (62.8%)	1.18 (0.56–2.48)	0.66	1.36 (0.55–3.37)	0.51
Antipsychotics^a^	35/55 (63.6%)	110/169 (65.1%)	0.94 (0.50–1.77)	0.84	0.38 (0.14–1.09)	0.07
Benzodiazepine^a^	62/89 (70.0%)	83/135 (61.5%)	1.44 (0.81–2.54)	0.21	1.83 (0.86–3.90)	0.12
SSRI^a^	1/3 (33.3%)	144/221 (65.2%)	0.27 (0.02–3.00)	0.28	0.11 (0.01–2.23)	0.15
TCA	2/4 (50.0%)	143/220 (65.0%)	0.54 (0.07–3.90)	0.54	‐	‐
Other antidepressant	6/11 (54.6%)	139/213 (65.3%)	0.64 (0.19–2.16)	0.47	‐	‐
Glucocorticoids^a^	9/18 (50.0%)	136/206 (66.0%)	0.51 (0.20–1.35)	0.18	0.37 (0.11–1.26)	0.11
Anticoagulant^a^	36/66 (54.6%)	109/158 (69.0%)	0.54 (0.30–0.97)	0.04	0.31 (0.14–0.69)	0.0042
Depression^a^	3/5 (60.0%)	142/219 (64.8%)	0.81 (0.13–6.27)	0.82	2.10 (0.24–18.05)	0.5
Bipolar disorder^a^	1/3 (33.3%)	144/221 (65.2%)	0.27 (0.01–2.83)	0.27	0.49 (0.03–7.14)	0.6
Mental retardation^a^	6/9 (66.7%)	139/215 (64.7%)	1.09 (0.28–5.29)	0.9	0.71 (0.11–4.55)	0.72
Dementia^a^	49/80 (61.3%)	96/144 (66.7%)	0.79 (0.45–1.40)	0.42	0.89 (0.42–1.90)	0.76
Alcoholism^a^	6/7 (85.7%)	139/217 (64.1%)	3.37 (0.56–64.18)	0.21	‐	‐
Parkinson's disease^a^	4/9 (44.4%)	141/215 (65.6%)	0.42 (0.10–1.63)	0.21	0.39 (0.08–1.98)	0.25
Malignant tumor^a^	23/35 (65.7%)	122/189 (64.6%)	1.05 (0.50–2.31)	0.89	0.66 (0.26–1.69)	0.39
Gastrectomy^a^	4/6 (66.7%)	141/218 (64.7%)	1.09 (0.21–8.00)	0.92	0.90 (0.11–7.42	0.92
Osteoporosis^a^	28/46 (60.9%)	117/178 (65.7%)	0.81 (0.42–1.60)	0.54	0.88 (0.38–2.03)	0.76
Vulnerable fracture	24/41 (58.5%)	121/183 (66.1%)	0.72 (0.36–1.46)	0.36	‐	‐
Hyperthyroidism^a^	3/5 (60.0%)	142/219 (64.8%)	0.81 (0.13–6.27)	0.82	0.88 (0.12–6.36)	0.9
Diabetes mellitus^a^	33/48 (68.8%)	112/176 (63.6%)	1.26 (0.64–2.54)	0.51	1.39 (0.60–3.22)	0.43
CKD^a^	62/86 (72.1%)	83/138 (60.1%)	1.71 (0.96–3.10)	0.07	2.29 (1.04–5.03)	0.0388
Smoking^a^	29/45 (64.4%)	116/179 (64.8%)	0.98 (0.50–1.98)	0.96	0.98 (0.38–2.53)	0.97

Abbreviations: BMI, body mass index; CI, confidence interval; CKD, chronic kidney disease; OR, odds ratio; SSRI, selective serotonin reuptake inhibitors; TCA, tricyclic antidepressants.

^a^ Variables included in multivariate model.

The risk from Parkinson's disease did not significantly differ in either univariate or multivariate analysis (OR: 0.42, 95% CI: 0.10–1.63, *p* = 0.21 and OR: 0.39, 95% CI: 0.08–1.98, *p* = 0.25, respectively).

## DISCUSSION

To our knowledge, this is the first study to examine the association between femoral neck fracture displacement and psychiatric disorders, including schizophrenia. In this study, we showed that schizophrenia is related to the displacement of femoral neck fractures. No epidemiological study has investigated the mechanism of displacement in femoral neck fractures or identified the factors that affect displacement; however, it is clinically believed that the greater the external force applied at the time of fracture, the longer the time elapsed since fracture, and the greater the external force applied to the fracture site, the greater the displacement.

Reasons for the association of schizophrenia with femoral neck fracture displacement are discussed further.

### Impact of the fall

It is possible that large external forces are applied during falls in schizophrenia. As the schizophrenia group was younger and more active than the control group, it was assumed that the impact of falls was greater. Therefore, a greater external force was applied at the time of fracture. These results suggest that schizophrenia is associated with displacement. Cauley suggests that parkinsonism is related to non‐displacement intertrochanteric fractures (not femoral neck fractures, but the same as hip fractures), and accordingly, the slow walking speed of patients with parkinsonism may reduce the impact of falls.^25^ In many cases, patients with schizophrenia develop drug‐induced parkinsonism because of the side‐effects of antipsychotics. However, in our study, Parkinson's disease was not a displacement‐related factor for femoral neck fractures, and the influence of other factors was considered strong, suggesting that our analysis was able to exclude the influence of drug‐induced Parkinsonism.

### Low pain sensitivity

Patients with schizophrenia are considered to have low pain sensitivity, and there are many reports of reduced pain sensitivity in people with schizophrenia, as described by Kraepelin and Bleuler since the early 20th century.^26^, ^27^ Even if a fracture is present, the patient is unlikely to feel pain. It is thought that it takes time for the fracture to be identified, during which time tendon traction causes dislocation to progress. Furthermore, the displacement is more severe because the patient does not remain at rest owing to low pain sensitivity and continues to apply an external force to the fracture site, such as walking, despite the fracture.

### Cognitive decline

Displacement may be exacerbated by the cognitive decline in patients with schizophrenia. Strömberg et al. reported that cognitive function decline is related to fracture displacement in their study of hip fractures in patients aged less than 65 years.^28^ In contrast, we found no relationship between these variables in our study. The study design was different, and in Strömberg's study, older patients' medical histories, including schizophrenia, were not evaluated; therefore, it is hoped that further research will clarify this point.

### Poor bone quality

Poor bone quality in patients with schizophrenia may be associated with greater fracture displacement. Owing to the lifestyle of patients with schizophrenia, including physical activity, diet, and smoking, their bone quality is assumed to be poor.^29^, ^30^ Sugawara et al. reported that Japanese patients with schizophrenia had a lower bone mass than the general population.^31^ Tseng et al. found that bone mineral density was significantly lower in patients with schizophrenia than in healthy controls.^14^ Many patients with schizophrenia and femoral neck fractures have chronic residual conditions. Kishimoto et al. suggested that a lack of physical activity, malnutrition, smoking, alcohol use, and polydipsia may be associated with poor bone quality.^5^ In addition, there may be an iatrogenic reason for the displacement of femoral neck fractures. Antipsychotic medications, which schizophrenic patients with schizophrenia usually take for years, are associated with increased prolactin levels, which can cause osteoporosis.^4^

### Scarcity of medical resources

Problems with the social system in psychiatry, especially in single‐subject psychiatric hospitals, may be associated with exacerbation of fracture displacement in schizophrenia. In Japan, most patients with schizophrenia are long‐term inpatients admitted to single‐departmental psychiatric hospitals. Compared with general hospitals, single‐departmental hospitals have fewer medical resources. Night‐time X‐ray examinations are often unavailable, and blood tests are often outsourced. In many psychiatric faculties in Japan, there may be a lack of resources to perform appropriate radiographic examinations and treatment interventions. A problem with many healthcare systems is that psychiatry is not integrated into general medical settings.

### Problems on the physician's side

The poor ability of psychiatrists to treat physical illnesses may be related to displacement of schizophrenic fractures. In general, psychiatrists are less adept at diagnosing and treating physical illnesses, including fractures, as their psychiatric careers progress. The psychiatrist's skill may delay the detection of the fracture, during which time the displacement may be exacerbated. In addition, poor coordination between general and psychiatric hospitals may be related to the exacerbation of the dislocation of schizophrenic fractures. This finding may be related to the stigma associated with schizophrenia. In Japan, general hospitals do not have psychiatrists on their staff, and psychiatrists are often absent or work part‐time. After hospitalization, adverse events during and after medical intervention occur more frequently in patients with schizophrenia than in those without schizophrenia.^32^, ^33^ Patients with schizophrenia are often isolated and frequently fail to adhere to the recommendations of their doctors concerning treatment, and it can be assumed that they also have problems maintaining their treatment regimens for physical illnesses.^34^ Without the support of a psychiatrist, physical medicine physicians have difficulty dealing with patients with schizophrenia. Thus, patients with schizophrenia may take longer to adjust to acceptance at general hospitals, and their displacement may be exacerbated while waiting for adjustment.

### Clinical implication

Our study provides clinical implications for the treatment of femoral neck fractures in patients with schizophrenia, whose numbers are expected to increase in the future. If a patient with schizophrenia has an episode of suspected femoral neck fracture, such as a fall, the patient should not be judged solely based on clinical findings, such as signs of pain, but should be referred to an orthopedic surgeon for an early radiographic examination.

### Limitations

This study has several limitations. First, this was a retrospective study with a retrospective review of medical records, and there was the potential for unmeasured confounding factors. Second, these data were from a single institution, and the sample size was small; therefore, the results of this study may not be generalizable. However, it has been reported that the displacement rate of femoral neck fractures is approximately 60%–70% in most cases,^25^, ^35^ and the rate in our study was 64.7%; therefore, we believe that our sample is representative of the population. Third, this study used data from a Japanese population. Our data may not adequately reflect the medical circumstances in other countries or districts because of certain conditions in Japan, such as the inadequacy of medical treatment systems for psychiatric physical complications, long‐term hospitalization, tendency to prescribe excessive amounts, or too many types of antipsychotics. Therefore, further well‐designed prospective studies including bone turnover markers as intermediate endpoints are warranted to support our findings.

### More comprehensive exploration

Future research should explore the circumstances surrounding the occurrence of femoral neck fractures. It would be beneficial to investigate, using rating scales, whether patients with schizophrenia who experience displacement with a femoral neck fracture exhibit more severe mental status compared to patients without displacement. In the case of patients with psychiatric and physical complications, it is thought that diagnosis and treatment are often carried out through several hospitals; it is important to collect detailed data on both psychiatric and physical aspects with collaboration between them.

## CONCLUSION

We found an association of schizophrenia with the displacement of femoral neck fractures. Based on our conclusions, it is recommended that in clinical situations, when a patient with schizophrenia falls, even in the absence of clear signs of pain, a femoral fracture should be suspected and thorough physical examination should be performed, including radiographic examination if necessary. In the case of a fracture, surgery should be performed without delay for the benefit of the patient.

## AUTHOR CONTRIBUTIONS

**Yukiyo Inoue**: Conceptualization; methodology; data curation; formal analysis; investigation; writing—original draft. **Akihiro Tokushige**: Methodology; formal analysis; supervision. **Takeshi Kinjyo**: Data curation; investigation. **Shinichiro Ueda**: Methodology; supervision.

## CONFLICT OF INTEREST STATEMENT

The authors declare no conflicts of interest.

## ETHICS APPROVAL STATEMENT

This study was approved by the Ethics Committee of the NMC and was conducted in accordance with the ethical guidelines for clinical research of the Ministry of Health, Labour, and Welfare, Japan, and the Declaration of Helsinki.

## PATIENT CONSENT STATEMENT

Informed consent was obtained from all participants on an opt‐out basis.

## CLINICAL TRIAL REGISTRATION

N/A.

## Supporting information


**Supporting Information**.

## Data Availability

The data that support the findings of this study are available from the corresponding author upon reasonable request.
