# Supplementary material for: Association of schizophrenia with fracture‐related femoral neck displacement: A cross‐sectional retrospective study
Source: PCN Rep. 2024 May 6;3(2):e195. doi: 10.1002/pcn5.195 (PMC11114322; doi:10.1002/pcn5.195)
Supplement: Supplementary file 1 — Supporting Information. [file PCN5-3-e195-s001.docx]

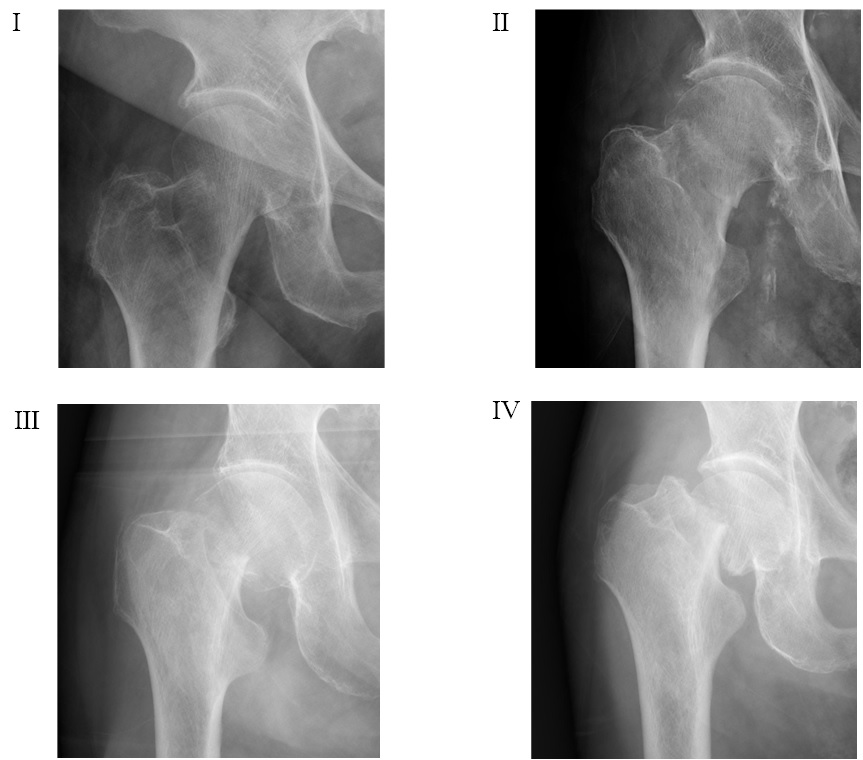


Supplemental Fig 1. Garden classification of Femoral Neck Fractures

Stage I: Incomplete fracture. The medial side of the neck is undamaged.

Stage II: Complete fracture without displacement. The upper and lower parts of the fracture are stuck.

Stage III: Complete fracture with partially rotated displacement. There is continuity of the medial femoral rotational artery.

Stage IV: Complete fracture with complete displacement. The medial femoral rotator artery is ruptured.

Garden stage I and II are the non-displaced type, and Garden stage III and IV are the displaced type.
